# Hepatic expression of cholesterol regulating genes favour increased circulating low-density lipoprotein in HIV infected patients with gallstone disease: a preliminary study

**DOI:** 10.1186/s12879-021-05977-0

**Published:** 2021-03-23

**Authors:** Suman Mewa Kinoo, Anil A. Chuturgoon, Bugwan Singh, Savania Nagiah

**Affiliations:** 1 Department of Medical Biochemistry, School of Laboratory Medicine and Medical Science, College of Health Science, University of KwaZulu Natal, Durban, Glenwood 4041 South Africa; 2 Discipline of General Surgery, School of Clinical Medicine, College of Health Science, University of KwaZulu Natal, Umbilo, Durban, 4001 South Africa; 3 Present address: Department of Human Biology, Medical Programme, Faculty of Health Sciences, Nelson Mandela University Missionvale Campus, Room 113, 2nd floor, Road, Salt Pan, Bethelsdorp, Port Elizabeth, 6059 South Africa

**Keywords:** Gall stone, Cholelithiasis, HIV, Cholesterol, LDLr, ABCA1, miR-148a

## Abstract

**Background:**

HIV endemic populations are displaying higher incidence of metabolic disorders. HIV and the standard treatment are both associated with altered lipid and cholesterol metabolism, however gallstone disease (a cholesterol related disorder) in Sub-Saharan African populations is rarely investigated.

**Methods:**

This study sought to evaluate hepatic expression of key genes in cholesterol metabolism (*LDLr*, *HMGCR*, *ABCA1*) and transcriptional regulators of these genes (microRNA-148a, *SREBP2*) in HIV positive patients on antiretroviral therapy presenting with gallstones. Liver biopsies from HIV positive patients (cases: *n* = 5) and HIV negative patients (controls: n = 5) were analysed for miR-148a and mRNA expression using quantitative PCR.

**Results:**

Circulating total cholesterol was elevated in the HIV positive group with significantly elevated LDL-c levels(3.16 ± 0.64 mmol/L) relative to uninfected controls (2.10 ± 0.74 mmol/L; *p* = 0.04). A scavenging receptor for LDL-c, *LDLr* was significantly decreased (0.18-fold) in this group, possibly contributing to higher LDL-c levels. Transcriptional regulator of *LDLr*, *SREBP2* was also significantly lower (0.13-fold) in HIV positive patients. Regulatory microRNA, miR-148a-3p, was reduced in HIV positive patients (0.39-fold) with a concomitant increase in target *ABCA1* (1.5-fold), which regulates cholesterol efflux.

**Conclusions:**

Collectively these results show that HIV patients on antiretroviral therapy display altered hepatic regulation of cholesterol metabolizing genes, reducing cholesterol scavenging, and increasing cholesterol efflux.

## Background

Gallstones account for more than 95% of biliary tract diseases in developed countries affecting 20% of the population [1]. In South Africa, gallstones were considered an affliction predominantly in Caucasians, however there has been an exponential increase in gallstone disease in black South Africans over the past few decades [2], [3]. Metabolic disorders in developing countries are a growing concern, paralleled with an increase in human immune deficiency virus (HIV) and a consequent judicious roll out of anti-retroviral therapy (ART). Whilst a significant amount of work has linked HIV and ART to altered fat and cholesterol metabolism contributing to metabolic syndrome like effects, diabetes mellitus and cardiovascular disease [4–7], no studies have looked at gallstone disease in this population.

Gallstones can be categorised into 3 types - cholesterol, pigment or mixed, dependent on cholesterol concentration. Cholesterol stones account for ~ 80% of all gallstones [8] and is a result of cholesterol superseding its saturation point in bile, causing cholesterol microcrystal formation, inevitably leading to gallstone formation [9]. Demographic, environmental and genetic factors may all contribute to altered cholesterol homeostasis [10], presenting complex molecular events that favour gallstone formation. Cholesterol homeostasis is maintained by various endogenous responses involving cholesterol synthesis, transport and excretion [11, 12].

Cholesterol biosynthesis is regulated by the rate-limiting enzyme of the mevalonate pathway, 3-hydroxy-3-methyl-glutaryl-CoA (HMGCoA) reductase (HMGCR) [13]. Transcription of *HMGCR* is controlled by transcription factor sterol regulatory element-binding proteins (SREBP1/2) which in turn is regulated by liver X receptors (LXRα/β), both of which act as biological cholesterol sensors [14, 15]. The central role of LXR and SREBP transcription factors on lipid and cholesterol metabolism make these prime targets in treating metabolic disorders. Cellular regulation of cholesterol flux also plays a key role in maintaining circulating cholesterol levels - Low-density lipoproteins (LDL) are scavenged by LDL receptors (LDLr) while a major determinant of high-density lipoproteins (HDL) is efflux pumps belonging to the ATP-binding cassette (ABC) family including ABCA1. The genes coding for these proteins are considered lithogenic and are implicated in gallstone progression which can be altered during HIV infection [16]. Transcriptional regulation of gene expression offers an opportunity to identify molecular events influencing disease phenotype.

MicroRNAs (miRNAs), short non-coding RNA (18–24 nucleotides) are epigenetic modulators of gene expression. MicroRNA prevent translation of target messenger RNA (mRNA) by binding via base complementarity. Various miRNAs have been associated with metabolic disorders through their regulatory function on cholesterol and lipid metabolism genes [17]. The role of miRNA in gallstone disease is unclear, however some miRNAs have been identified as regulators of genes involved in gallstone formation. MicroRNA-122a and miR-144a directly inhibit cholesterol 7 alpha hydroxylase (*CYP7A1*) influencing the bile acid pool [12]. Yang et al. (2015) performed an integrated analysis of miRNA/mRNA networks in gallstones, identifying 17 differentially regulated miRNAs [18]. This study showed miRNAs that regulated lipoprotein signalling and ABC transporters were significantly altered in gallstone patients. MicroRNA-148a is a miRNA that is highly expressed in liver and associated with altered LDL-c and triglyceride levels in humans [19]. *LDLr* is shown to be directly regulated by miR-148a and is linked to gallstone formation in high fat diet fed mice [20].

Dysregulated miRNA in HIV infection are more well established than miRNA associated with biliary tract disorders, with several papers linking altered miRNAs to disease progression, metabolic outcomes, treatment responsiveness and biomarkers [21, 22]. Despite the established link of HIV and ART to metabolic disorders, there is a dearth of knowledge on gallstone disease in this population. The added effects of ART are known to alter cholesterol metabolism by various mechanisms. Protease inhibitors (PI’s) have the greatest dyslipidaemia effects. Nucleoside reverse transcriptase inhibitors (NRTI’s) and Non-Nucleoside reverse transcriptase inhibitors (NNRTI’s) have less dyslipidaemia effects, whilst Intergrase strand transfer inhibitors (InSTI’s) have a neutral effect [23]. In theory the observed metabolic changes in people living with HIV may contribute to cholesterol GD, as these patients are known to have altered circulating lipid levels. The evidence for this is that lipid levels change with HIV RNA levels independent of ART. In ART naïve patients, literature from the West (where HIV-1 subtype B is prevalent), report an increase in very low-density lipoprotein (VLDL), cholesterol (TC), and triglyceride (TG) levels with increasing HIV viral load and a decrease in high-density lipoprotein (HDL) and low-density lipoprotein (LDL) [24]. African’s normally display higher fasting HDL and lower triglyceride levels than Caucasians [25], however in ART naïve patients in South Africa where HIV-1 subtype C is most prevalent HDL levels were found to be lower which is seen as an increased risk for CVD and possibly cholesterol GD [26].

In Japan, studies report an increased rate of cholelithiasis (9,8%) in HIV positive patients on protease inhibitor (PI)-inclusive ART [27]. Lin et al. (2015) demonstrated that the accumulative exposure to atazanavir/ritonavir for over 2 years is associated with a 6.29-fold increase in the risk for incident cholelithiasis [28]. Another French study reported Indinavir induced cholelithiasis in one patient [29]. While these studies indicate the possibility of ART-associated cholelithiasis, these are based on PI-inclusive regimens, which are less frequently used in South Africa. Despite newer integrase inhibitor (II) ART drugs showing lower lipid abnormalities than previously used PI-based ART, abnormalities in lipid concentration still occur, and this may be reflective either of the viral effects itself, chronic ART use or the persistent inflammation and immune activation in these HIV infected patients [30].

## Methods

The present study sought to evaluate the hepatic expression of genes involved in cholesterol homeostasis (*LDLR*, *ABCA1*, *HMGCR*) in Black South African HIV positive patients presenting with gallstones relative to HIV negative patients with gallstone disease. Further, transcriptional regulators of these genes (*SREBP2*, miR-148a) were evaluated.

This study utilized a case-control design comparing hepatic expression of cholesterol homeostasis related miRNA in HIV positive (case) and HIV negative (control) patients presenting with symptomatic gall stones. Ethical approval was obtained from the University of KwaZulu Natal Biomedical Research Ethics Committee (BREC – BE276/16) and all subsequent methodologies were conducted in accordance with the guidelines provided by the Declaration of Helsinki. Patients undergoing cholecystectomy for gall stone disease (biliary cholic, acute cholecystitis, jaundice and gall stone pancreatitis) at King Edward VIII Hospital, Durban, KwaZulu Natal, South Africa from January – December 2017 were recruited to evaluate known risk factors of gall stone formation (age, oestrogen exposure, family history, obesity) in HIV positive and negative patients. Demographic data as per the self-identified questionnaire included age, ethnicity, 1st degree family history of gallstones, and HIV status, including the use or not of ART. Oestrogen exposure was defined based on pregnancies and history of contraception use and was assessed as a binary variable. BMI was measured by the main author using the same stadiometer for all patients to avoid measurement error. Patients undergoing cholecystectomy for reasons other than gallstones but where gallstones were an incidental finding were excluded from the study. Patients whose HIV-status was unknown or who refused testing after voluntary counselling were excluded. All patients had stones that macroscopically met the description of cholesterol stones. None of the patients had pigment-stones. None of the patients were on any lipid-lowering medication such as fibrates which predispose to gallstones. None of the patients were screened for any hepatitis viruses. Lipograms [including triglyceride, high density lipoprotein (HDL), low density lipoprotein (LDL) and total cholesterol] were performed on all patients in a fasting state and were measured directly by pathology testing lab, Ampath laboratories.

In total 96 patients gave informed consent (standard consent form in two official main languages English and isiZulu) for retrieval of a liver biopsy and recording of clinical parameters of patients including age, race, BMI, family history of gall stones and comorbidities (HIV, arterial hypertension, diabetes mellitus, hypercholesterolemia).

Following the analysis of clinical parameters, five HIV negative (control) and five HIV positive (cases) were selected for miRNA and downstream target analysis. Inclusion criteria included women of Black African ethnicity between the ages of 18–50, presented with symptomatic cholelithiasis, were all on contraceptive for more than 1 m, and did not have a family history of gallstones. Patients with co-morbidities (diabetes mellitus, arterial hypertension, hypercholesterolemia, chronic inflammatory disorders), receiving chronic therapy for the above-mentioned co-morbidities and HIV positive patients on preventative TB therapy were excluded. All HIV positive patients were on fixed dose combination therapy with CD4 counts above 500 cells/mm^3^ and undetectable viral loads.

### RNA extraction

Briefly, liver tissue (1cmx1cm) was submerged in RNAlater® Stabilization Reagent (Qiagen, Hilden, Germany) in 2 ml cryovials at collection and stored at − 80 °C until RNA extraction. The stabilization buffer was decanted, and tissue samples were rinsed in 0.1 M phosphate saline buffer (PBS) prior to RNA extraction via the Qiazol extraction method as per the manufacturer’s instructions. Crude RNA was quantified using the Nanodrop 2000 spectrophotometer (Thermofisher Scientific, Waltham, MA, USA) and 1 μg of RNA was used for complementary DNA (cDNA) synthesis. The miScript RT II Kit (Qiagen) was used for miRNA quantification and the QuantiTect Reverse Transcription kit (Qiagen) was used for mRNA quantification as per the manufacturer’s instructions.

### MicroRNA-148a and mRNA quantification

Hepatic expression of microRNA-148a in patients was measured using a miScript Primer Assay specific for human miR-148a-3p (Qiagen) as per the manufacturer’s instructions. Briefly, a reaction volume of 25 μl consisting of template cDNA, 2X QuantiTect SYBR Green PCR Master Mix, 10X miScript Universal Primer, 10X miScript Primer Assay and nuclease free water was prepared in a MicroAMP™ 96-Well Base plate (Applied Biosystems, Foster City, CA, USA) and sealed. Thermocycler conditions for quantitative PCR were as follows - Initial denaturation (95 °C, 15 min) followed by 40 cycles of denaturation (94 °C, 15 s), annealing (55 °C, 30 s) and extension (70 °C. 30 s, fluorescence reading). House-keeping gene RNU6 was concurrently quantified for normalization of gene expression. Thermocycler conditions and data capturing were performed using the Applied Biosystems Viia7 Real-Time PCR System.

Messenger RNA quantification was performed using the PowerUp™ SYBR™ Green Master Mix (ThermoFisher) system as per the manufacturer’s instructions. A reaction volume of 10 μl was made up consisting of 5 μl PowerUp® SYBR® Green Master Mix, 1 μM of sense and antisense primers and nuclease free water. Thermocycler conditions were as follows: 50 °C 2 min, initial denaturation (95 °C, 15 s), and 40 cycles of denaturation (95 °C, 15 s), annealing Table 1, (15 s), and extension (72 °C, min). Housekeeping genes *GAPDH* and *18S* were concurrently quantified for normalization of results. Thermocycler conditions and data capturing were performed using the Applied Biosystems Viia7 Real-Time PCR System.

**Table 1:** Clinical parameters of patients presenting with gall stones (HIV negative vs HIV positive)

	Control *n* = 5; Mean ± SD	HIV *n* = 5; Mean ± SD	*P*-value
Age (years)	30 ± 5.41	40 ± 6.19	0.027
BMI (kg/m^2^)	34.06 ± 5.98	32.63 ± 10.84	0.81 (ns)
Total Cholesterol (mmol/L)	3.64 ± 1.11	4.88 ± 0.988	0.099 (ns)
Triglycerides (mmol/L)	0.76 ± 0.389	0.90 ± 0.49	0.65 (ns)
HDL (mmol/L)	1.19 ± 0.28	1.33 ± 0.58	0.65 (ns)
LDL (mmol/L)	2.10 ± 0.74	3.16 ± 0.64	0.04
CD4 count (cells/μl)	n/a	460 ± 125	
ART duration (years)	n/a	4 ± 2.6	

*ns no significance

All primer sequences were obtained using the search engine Primer Bank (https://pga.mgh.harvard.edu/primerbank/) using the keyword search function. Subsequently, selected primer sequences were run through NCBI Basic Local Alignment Search Tool (BLAST) to test for specificity.

### Statistical analysis

Comparisons of clinical parameters between HIV negative (control) and HIV positive patients (cases) presenting with gall stones were made by performing a Mann-Whitney U two-tailed Test using Prism 7 statistical software (GraphPad Prism Inc., La Jolla, CA, USA). *P* values < 0.05 were considered statistically significant.

Quantitative PCR analysis of miRNA and mRNA levels was performed using QuantStudio 7 Pro Real-Time PCR Systems Software (Thermofisher). The software reports microRNA and mRNA levels as fold change relative to the control calculated s 2^-ΔΔCt^ (RQ min; RQ max) and changes > 2 and < 0.5 are considered significant. This is the standard for the Livak method of calculating fold change from Ct values [31].

## Results

Analysis of clinical parameters are summarized in Table 2. The HIV positive group was significantly older than the HIV negative controls. The results show that the HIV positive group had a lower BMI with overall higher levels of total cholesterol, triglycerides, high density lipoprotein cholesterol (HDL-c) and significantly higher low-density lipoprotein cholesterol (LDL-c).

**Table 2:** Primer sequences and annealing temperatures for quantitative PCR

GENE	PRIMER SEQUENCE	ANNEALING TEMPERATURE (°C)
*LDLR* forward *LDLR* reverse	5′-GAGAGCTTGTGCCGAGATGTG-3′ 5′- CCGCAGTTGTTAGTGCCATCA-3′	58
*SREBP2* forward *SREBP2* reverse	5′- CCTGGGAGACATCGACGAGAT-3′ 5′- TGAATGACCGTTGCACTGAAG-3’	54
*HMGCR* forward *HMGCR* reverse	5’- TGATTGACCTTTCCAGAGCAAG-3′ 5′- CTAAAATTGCCATTCCACGAGC-3’	53
*ABCA1* forward *ABCA1* reverse	5’-GGAAGAACAGTCATTGGGACAC-3′ 5′- GCTACAAACCCTTTTAGCCAGT-3’	58

None of the patients were on any voluntary diets or exercise regimens. All HIV positive patients had undetectable viral loads and were on fixed dose combination (FDC) ART. FDC regimen consisted of two NRTIs - Tenofovir Disoproxil Fumarate (TDF) and Emtricitabine (FTC), and one NNRTI - Efavirenz (EFV).

### microRNA-148-3p and target gene quantification

**Fig. 1:**
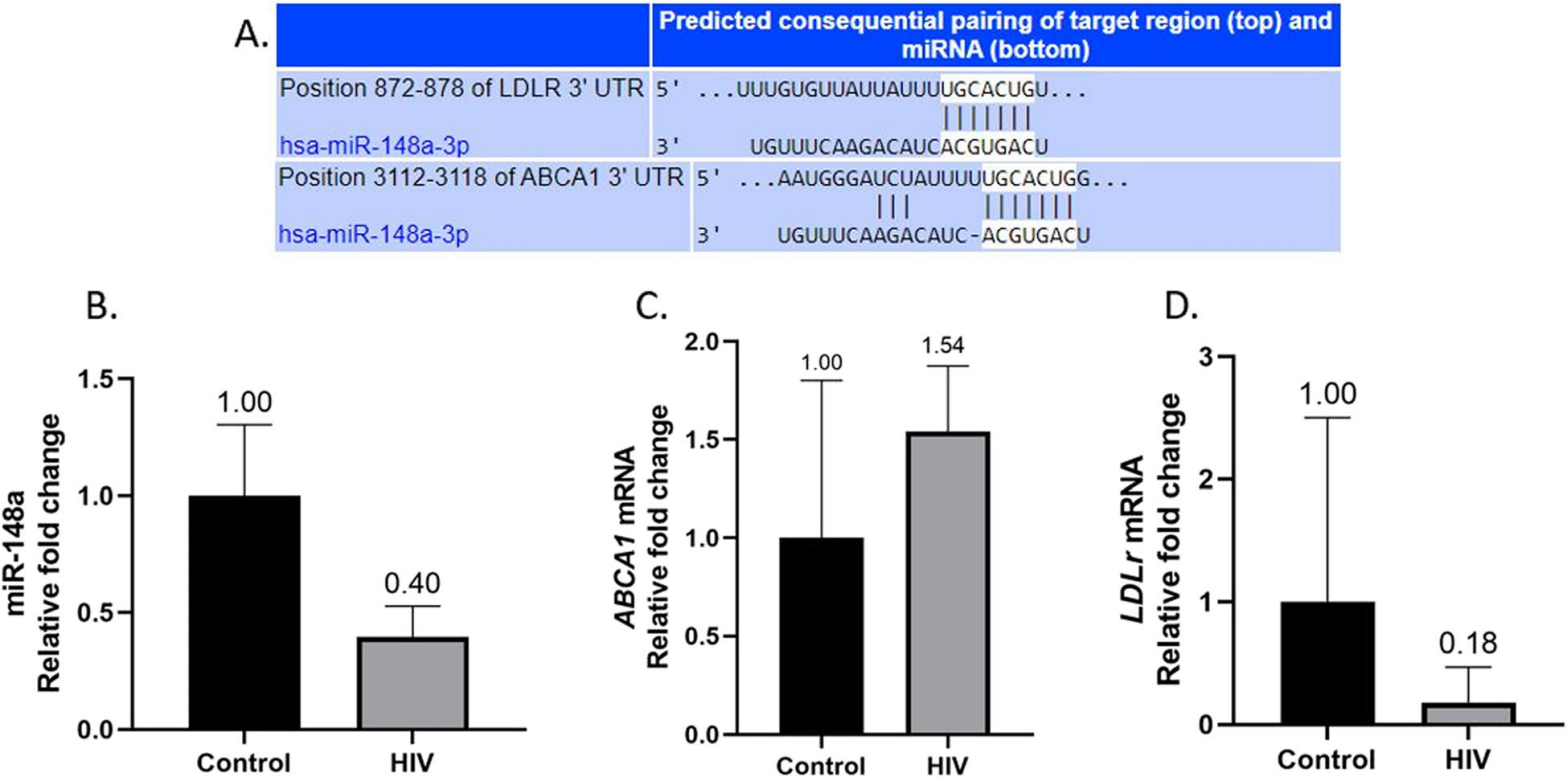
MicroRNA-148-3p is a regulator of cholesterol metabolism through its binding capacity on *ABCA1* and *LDLr* **a**. Hepatic expression is significantly lower in HIV positive patients **b** with an inverse observation made with *ABCA1* mRNA levels **c**. *LDLr* mRNA levels were significantly lower in HIV infection **d**

Gene targets of miR-148-3p were confirmed using TargetScan prediction software confirming complementary base pairing with *ABCA1* and *LDLr* (Fig. 1a). Quantitative PCR results for miR-148a-3p showed significantly lower hepatic expression in HIV positive individuals [0.396-fold (RQ min:0.297; RQ max: 0.527); Fig. 1b]. Analysis of target gene *ABCA1* showed higher mRNA levels [1.541-fold (RQ min: 1.266; RQ max: 1.875); Fig. 1c] while *LDLr* mRNA levels were significantly reduced in HIV positive patients [0.181-fold (RQ min: 0.07; RQ max: 0.467); Fig. 1d].

### Transcriptional regulation of LDLR via SREBP2

**Fig. 2:**
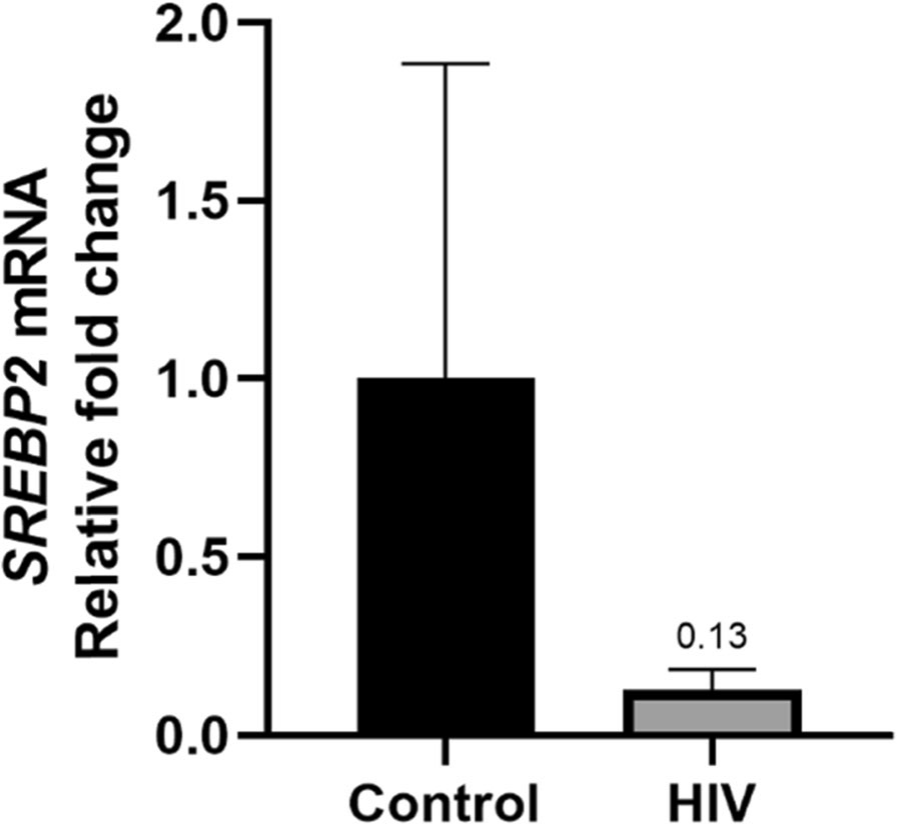
Quantitative PCR analysis of *SREBP2* mRNA showed significantly lower expression in HIV positive patients relative to HIV negative controls

Following analysis of miR-148a-3p and target genes *ABCA1* and *LDLr*, alternate transcriptional regulation of *LDLr* was investigated to justify the significantly lower mRNA levels observed in HIV infected individuals. Quantitative PCR results showed significantly lower *SREBP2* (transcriptional regulator of *LDLr*) mRNA levels in HIV positive patients presenting with gall stones compared to HIV negative patients [0.127-fold (RQ min: 0.088; RQ max: 0.184); Fig. 2].

### Cholesterol metabolism

**Fig. 3:**
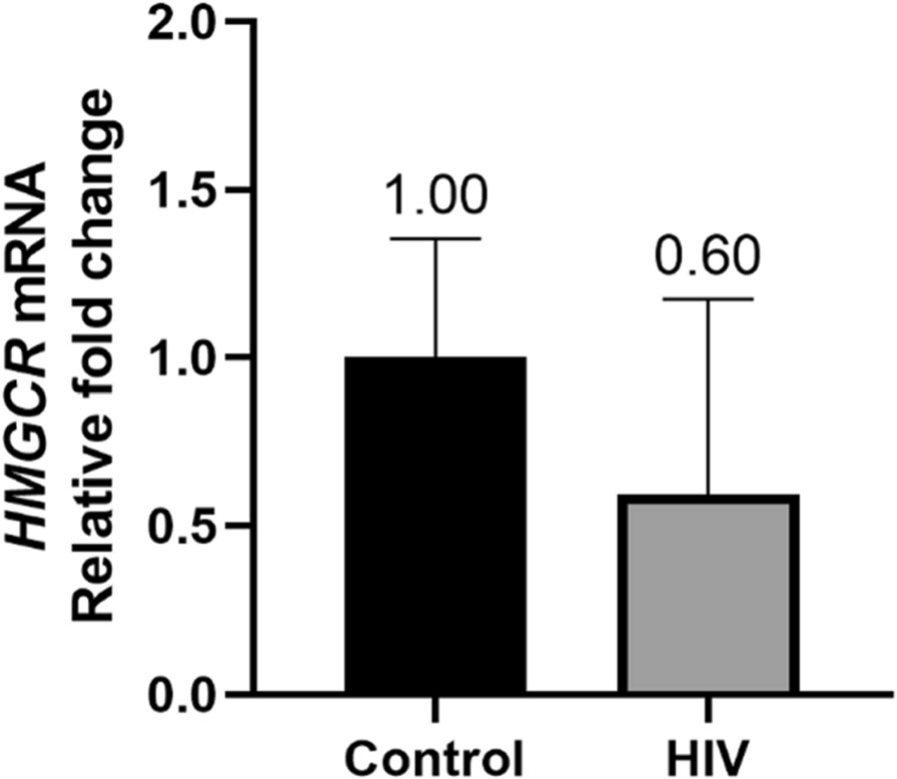
Quantitative PCR analysis showed 0.6-fold change in *HMGCR* mRNA levels in HIV positive patients relative to HIV negative controls

HIV positive patients showed higher levels of total cholesterol, therefore the gene responsible for cholesterol metabolism was quantified. *HMGCR* mRNA levels were lower in the HIV positive group, albeit insignificantly [0.595-fold RQ min: 112; RQ max: 1.172); Fig. 3].

## Discussion

Urbanised diets and the consequent rise in obesity amongst Black South Africans has led to gallstones becoming more prevalent amongst this population group in recent years. Established risk factors for gallstones – over 40 years of age, female, pregnancy, overweight, high-fat diet, and being sedentary, are all lifestyle or demographic dependent [10]. Little is known on the implications of chronic infectious diseases like HIV on gallstone disease risk.. The present study sought to investigate molecular events that may contribute to this phenotype by measuring the hepatic expression of cholesterol metabolism genes that influence gallstone formation. Biological response to a high fat diet, the rate of cholesterol absorption, hepatic cholesterol metabolism and the concentration of cholesterol excreted in bile differs between patients with and without gallstones [32]. Raised LDL-c in circulation is strongly implicated in lithogenic states. Lowered HDL-c levels are also implicated in gallstone formation but data regarding this finding has been conflicting in reports [33].

Mounting evidence suggests the resultant abnormalities are linked to dietary and genetic factors. LDL-c is cholesterol that is available for delivery and cellular uptake; the circulating concentration is linked directly to dietary cholesterol consumption and associated with both cardiovascular disease and cholesterol gallstone formation [11, 34]. HDL-c, excess cholesterol transported from cells to the liver for excretion in a process called reverse cholesterol transport, is considered the “healthier” cholesterol due to protective properties against atherosclerosis. Lower HDL-c is linked to poorer cardiovascular outcomes and is also implicated in cholesterol gallstone formation [35]. Elevated activity of cholesteryl ester transfer protein (CETP) in patients with gallstones may lower HDL-c and increase LDL-c by transferring the lipoprotein from HDL to LDL [36].

A high circulating LDL-c will result in higher concentrations of cholesterol being delivered to the liver for excretion resulting in supersaturation of bile, inevitably leading to gallstone formation. This is best explained by Admirand’s triangular relationship between cholesterol, bile salts and lecithin, where biliary cholesterol hypersecretion supersedes the concentration of bile salts and lecithin with resultant precipitation and formation of cholesterol stones [9]. The cholesterol hypersecretion is due to excess free cholesterol pooling in the liver either due to increased HMGCR activity or larger volumes of cholesterol returning to the liver. Cholesterol is returned to the liver via one of 4 pathways; via the LDLr, via apoE-rich lipoproteins through the LDL receptor-like protein (LRP), via HDL_2_ free cholesterol “exchange,” or via nonreceptor-mediated LDL uptake [37].

Altered cholesterol metabolism, favouring LDL-c is complicit in gallstone formation, among other metabolic disorders such as cardiovascular disease. Although HIV and ART are linked to metabolic disorders leaning toward this phenotype, little is known of the effects of HIV on gallstone formation. In HIV positive patients, low levels of HDL-c are congruent with high viral loads, linking this metabolic profile directly to the virus itself independent of ART [24]. The use of ART reverses these effects. LDL-c levels however appear similar to HIV negative female patients, but rises dependant on the use of PI-ART [38]. In male patients however, there is an inverse relation with LDL-c and HIV RNA levels independent of ART, and this may well be a major contributing factor to males being relatively protective against gallstones as indicative of the low incidence of gallstones in HIV positive males [39].

These findings are not dissimilar to that in our study group where all patients were female and all on ART. The use of ART may explain the significantly higher LDL-c, and the slight increase in HDL-C in the HIV group compared to the control (Table 2). Altered circulating LDL-c would prompt cellular homeostatic responses. As HIV positive women in our study displayed higher LDL-c, we evaluated the first line of hepatic LDL-c uptake. Located on the cell membrane, LDLr is responsible for hepatic absorption of LDL, with higher expression reducing circulating LDL-c levels. The expression of *LDLr* is regulated by various regulatory mechanisms including transcription factors (SREBP2 and LXR) and epigenetic regulators like miR-148a. SREBP transcription factors regulate the biosynthetic pathway of cholesterol and *LDLr* by stimulating transcriptional genes containing sterol response elements [40]. There are 3 isoforms; *SREBP1a, SREBP1c* and *SREBP2. SREBP2* is highly expressed in liver amongst other cells. Abnormalities in these regulatory elements (decreased *SREBP2* and *LXR*) results in decreased *LDLr* expression and decreased LDL catabolism resulting in raised LDL-c serum levels [41].

HIV-1 infection of CD4^+^ T cells stimulates cholesterol biosynthesis via *SREBP2* sterol response gene (protein TFII-I) activation for enhanced HIV-1 transcription and HIV-1 replication [42]. HIV-1 transcription is thus modulated by LDL-c, since uptake of LDL-c results in feedback inhibition of *SREBP2*- dependent proteins such as TFII-I [42]. The hepatic impact of this in HIV patients with gallstones in not known. However, *LDLr* levels in mononuclear cells of both the blood and liver have been found to be down-regulated in HIV positive patients with lipodystrophy compared to HIV negative controls and positive patients without lipodystrophy, independent of PI-ART [43]. The pathogenesis of this lipodystrophy by down-regulation of *LDLr* may not be dissimilar to that of gallstone pathogenesis in HIV positive patients as these mimic our findings in this study. Further to this we demonstrated that the downregulation of *LDLr* (Fig. 1d) was due to a decrease in *SREBP2* mRNA (Fig. 2) with overall significantly raised LDL-c levels in the HIV group.

Cholesterol reverse transport is also an important determinant in cholesterol homeostasis. In this regard, the ABC family of efflux pumps plays a significant role in cellular efflux of cholesterol. ABCA1 is a major regulator of cellular reverse cholesterol transport by transporting cholesterol, mainly the lipid poor apoA1, out of the cell and converts it into mature HDL for transport back to the liver. Overexpression of *ABCA1* in transgenic mice results in a lithogenic state by increasing plasma HDL-c levels, hepatic delivery of HDL cholesteryl-esters and biliary cholesterol concentrations [44]. Its lithogenic role is further accentuated in gallbladder epithelial cells, where *ABCA1* is regulated by *LXR* and *RXR* and modulates biliary cholesterol concentrations and its excretion [45].

*ABCA1* expression can be epigenetically regulated by miR-148a. Inhibition of miR-148a increases *ABCA1* mRNA levels resulting in increased cholesterol efflux to ApoA1, thus increasing plasma HDL-c [20]. The inverse relationship between *ABCA1* by miR-148a is evident in HIV positive patients with gallstones compared to their negative counterparts as demonstrated in our study (Fig. 1b, c). The effect on plasma HDL-c however was negligible in our study (Table 2).

The effect of HIV on hepatic *ABCA1* expression has not been studied, however in order for survival and replication of the virus within lymphocytes, it requires large amounts of cholesterol within the cell. It achieves this by directly inhibiting *ABCA1*. The level of inhibition of cholesterol efflux is directly proportional to the level of viral replication within the cell. It achieves this by encoding a small protein called Negative Regulatory Factor (Nef) which binds to *ABCA1* and down regulates it thus preventing the efflux of apoA1 cholesterol to HDL [5].

Besides its regulatory role of *ABCA1*, miR-148a is considered a central miRNA in cholesterol and fat metabolism. MicroRNA-148a is located in a gene-poor intergenic region of human chromosome 7 and is predominantly expressed in liver [20]. Notably, the expression of miR-148a is significantly increased in the liver of high-fat diet (HFD)-fed mice [20]. Out of 159 miRNAs identified to be highly expressed in human liver and modulated by dietary lipids, miR-148a emerged as the strongest with highest liver activity and expression in livers of HFD fed mice [46]. Lastly, we assessed cholesterol biosynthesis in HIV positive patients via *HMGCR* levels. *HMGCR*, the rate limiting enzyme in the mevalonate pathway is regulated by *SREPB2* which up-regulates cholesterol synthesis genes when cholesterol levels are low [47]. Our study showed the HIV group having lower *HMGCR* mRNA levels (Fig. 3) which may be due to cholesterol levels being higher in this group.

There is a paucity of research on hepatic cholesterol-regulating gene expression in people with HIV on ART. Sopeyin et al. (2019) measured similar parameters, albeit in lymphocytes from HIV infected individuals on a TDF/EFV containing regimen – ABCA1 and HMGCR expression was increased in the patients on ART compared to those not on ART [48]. The efflux activity of ABCA1 is likely to then promote circulating levels of cholesterol. The same group published in vitro findings that EFV and TDF altered expression of 11 genes related to lipid and cholesterol biosynthesis, however a combination of both drugs did not significantly alter cholesterol biosynthesis [49]. Similarly, in our study *HMGCR*, the rate limiting enzyme on cholesterol biosynthesis was not significantly changed (Fig. 3). A more recent study by Pokora et al. (2020) evaluated the effect of TFV on LDL and LDLR expression in circulation in patients with chronic hepatitis B [50]. This study showed decreased levels of LDLR in patients receiving TFV compared to those that were not on TFV treatment. Again, this is similar to our study where LDLR expression in the liver is reduced in HIV patients on ART compared to uninfected individuals (Fig. 1d). It is however unfeasible to extrapolate the findings in literature on the effect of ART on the genes we investigated due to variations in the samples used, and most importantly, the different cell types evaluated.

The impact of HIV on non-communicable diseases, particularly related to disruptions in metabolism, is a growing concern in developing countries. While this study evaluated a very small sample size, differences in the expression of cholesterol regulating genes at the hepatic level were identified between HIV positive and HIV negative patients with the same disease. There is a dearth of knowledge on gallstone disease and HIV co-morbidities in Black African populations. Our findings show alterations to cholesterol transporter expression in patients with HIV and on ART, offering an insight into the pathology in this population. At a clinical level, it would be interesting to correlate these findings to circulating levels of these genes and their regulators for potential biomarker discovery. Further, identifying differentially expressed cholesterol regulating genes offers the unique opportunity of highly targeted interventions via negative gene regulators. The identification of miR-148a being differentially regulated in HIV positive patients is of special interest, as this can be investigated due to its effect on downstream targets and fluctuations in cholesterol flux.

The most obvious limitation of the study is the small sample number. Ideally the study would include samples from patients without gallstone disease and patients not on ART. These demographics proved difficult to collect in sufficient numbers during the patient recruitment period. Another limitation was the large average age gap between the HIV negative and HIV positive groups, which is likely to affect the observed results. The study could be enhanced by correlating the expression of these genes in the liver with circulating levels of the genes and lipogram data – these statistics would be more powerful with a bigger sample size. Furthermore, discrepancies in the duration of ART in the patients may lead to variability.

## Conclusion

Cholesterol homeostasis is complex, and dysregulation of the regulatory processes involved can lead to cholesterol gallstone formation. The impact of a chronic infectious disease, like HIV, needs to be considered in the context of rising incidence of metabolic disorders in developing countries. Our findings show a significant increase in circulating LDL-c in the HIV positive group coupled with reduced mRNA expression of hepatic *LDLr*. However, the suppression of miR-148, an epigenetic regulator of *LDLr*, was downregulated in the HIV group. This would indicate a possible alternate pathway in the downregulation of *LDLr* in HIV positive patients linked with raised LDL-c and gallstone formation and will require further investigation. MiR-148a however did appear to regulate *ABCA1* with an inverse relationship being observed in the HIV positive patients.

## Data Availability

The datasets used and/or analysed during the current study are available from the corresponding author on reasonable request.

## Abbreviations

ABC: Adenosine triphosphate binding cassette
ABCA1: Adenosine triphosphate binding cassette A1
ART: Antiretroviral therapy
BMI: Body mass index
cDNA: Complementary DNA
CETP: Cholesterol ester transfer protein
CYP7A1: Cholesterol-7 alpha-hydroxylase
GAPDH: Glyceraldehyde-3-phosphate dehydrogenase
HDL: High density lipoprotein
HDL-c: High density lipoprotein cholesterol
HMG CoA: 3-hydroxy-3-methyl-glutaryl-CoA
HMGCR: 3-hydroxy-3-methyl-glutaryl-CoA Reductase
HIV: Human Immunodeficiency Virus
LDL: Low density lipoprotein
LDL-c: Low density lipoprotein cholesterol
LDLr: Low density lipoprotein receptor
LRP: Low density lipoprotein receptor like protein
LXR: Liver X Receptor
miRNA: microRNA
mRNA: Messenger RNA
PBS: Phosphate saline buffer
PI: Protease inhibitor
RNA: Ribose nucleic acid
SREBP: Sterol regulatory element binding protein

## References

[1] Goldacre MJ, Duncan ME, Griffith M, Davidson M (2011). Trends in mortality from appendicitis and from gallstone disease in English populations, 1979–2006: study of multiple-cause coding of deaths. Postgrad Med J.

[2] Parekh D, Lawson HH, Kuyl JM (1987). Gallstone disease among black south Africans. S Afr Med J.

[3] Walker ARP, Segal I, Posner R, Shein H, Tsotetsi NG, Walker AJ (1989). Prevalence of gallstones in elderly black women in Soweto, Johannesburg, as assessed by ultrasound. Am J Gastroenterol.

[4] Cui HL, Grant A, Mukhamedova N, Pushkarsky T, Jennelle L, Dubrovsky L, Gaus K, Fitzgerald ML, Sviridov D, Bukrinsky M (2012). HIV-1 Nef mobilizes lipid rafts in macrophages through a pathway that competes with ABCA1-dependent cholesterol efflux. J Lipid Res.

[5] Mujawar Z, Rose H, Morrow MP, Pushkarsky T, Dubrovsky L, Mukhamedova N (2006). Human immunodeficiency virus impairs reverse cholesterol transport from macrophages. PLoS Biol.

[6] Rappocciolo G, Jais M, Piazza P, Reinhart TA, Berendam SJ, Garcia-Exposito L (2014). Alterations in cholesterol metabolism restrict HIV-1 trans infection in nonprogressors. MBio..

[7] Feeney ER (2011). HIV and HAART-associated dyslipidemia. Open Cardiovasc Med J.

[8] Acalovschi M (2001). Cholesterol gallstones: from epidemiology to prevention. Postgrad Med J.

[9] Admirand WH, Small DM (1968). The physicochemical basis of cholesterol gallstone formation in man. J Clin Invest.

[10] Goktas SB, Manukyan M, Selimen D (2016). Evaluation of factors affecting the type of gallstone. Indian J Surg.

[11] Dietschy JM, Turley SD, Spady DK (1993). Role of liver in the maintenance of cholesterol and low density lipoprotein homeostasis in different animal species, including humans. J Lipid Res.

[12] Li T, Francl JM, Boehme S, Chiang JYL (2013). Regulation of cholesterol and bile acid homeostasis by the cholesterol 7 a -hydroxylase/steroid response element-binding protein 2/microRNA-33a Axis in mice. Hepatology..

[13] Lindgren V, Luskeyt KL, Russellt DW, Francke UTA (1985). Human genes involved in cholesterol metabolism : chromosomal mapping of the loci for the low density lipoprotein receptor and 3-hydroxy-3-methylglutaryl-coenzyme a reductase with cDNA probes. Proc Natl Acad Sci U S A.

[14] Yoshikawa T, Shimano H, Amemiya-kudo M, Yahagi N, Hasty AH, Matsuzaka T (2001). Identification of liver X receptor-retinoid X receptor as an activator of the sterol regulatory element-binding protein 1c gene promoter. Mol Cell Biol.

[15] Repa JJ, Liang G, Ou J, Bashmakov Y, Lobaccaro JA, Shimomura I, Shan B, Brown MS, Goldstein JL, Mangelsdorf DJ et al. Regulation of mouse sterol regulatory by oxysterol receptors , LXR ␣ and LXR ␤. Genes Dev 2000;14:2819–2830, Regulation of mouse sterol regulatory element-binding protein-1c gene (SREBP-1c) by oxysterol receptors, LXRalpha and LXRbeta, 22, DOI: 10.1101/gad.844900. 10.1101/gad.844900 PMC317055 11090130

[16] Hernández-Nazará A, Curiel-López F, Martínez-López E, Hernández-Nazará Z, Panduro A (2006). Genetic predisposition of cholesterol gallstone disease. Ann Hepatol.

[17] Sud N, Taher J, Su Q (2016). HHS public access. Drug Dev Res.

[18] Yang B, Liu B, Bi P, Wu T, Wang Q, Zhang J (2015). An integrated analysis of differential miRNA and mRNA expressions in human gallstones. Mol BioSyst.

[19] Do R, Willer CJ, Schmidt EM, Sengupta S, Gao C, Peloso GM, Gustafsson S, Kanoni S, Ganna A, Chen J, Buchkovich ML, Mora S, Beckmann JS, Bragg-Gresham JL, Chang HY, Demirkan A, den Hertog HM, Donnelly LA, Ehret GB, Esko T, Feitosa MF, Ferreira T, Fischer K, Fontanillas P, Fraser RM, Freitag DF, Gurdasani D, Heikkilä K, Hyppönen E, Isaacs A, Jackson AU, Johansson Å, Johnson T, Kaakinen M, Kettunen J, Kleber ME, Li X, Luan J', Lyytikäinen LP, Magnusson PKE, Mangino M, Mihailov E, Montasser ME, Müller-Nurasyid M, Nolte IM, O'Connell JR, Palmer CD, Perola M, Petersen AK, Sanna S, Saxena R, Service SK, Shah S, Shungin D, Sidore C, Song C, Strawbridge RJ, Surakka I, Tanaka T, Teslovich TM, Thorleifsson G, van den Herik EG, Voight BF, Volcik KA, Waite LL, Wong A, Wu Y, Zhang W, Absher D, Asiki G, Barroso I, Been LF, Bolton JL, Bonnycastle LL, Brambilla P, Burnett MS, Cesana G, Dimitriou M, Doney ASF, Döring A, Elliott P, Epstein SE, Eyjolfsson GI, Gigante B, Goodarzi MO, Grallert H, Gravito ML, Groves CJ, Hallmans G, Hartikainen AL, Hayward C, Hernandez D, Hicks AA, Holm H, Hung YJ, Illig T, Jones MR, Kaleebu P, Kastelein JJP, Khaw KT, Kim E, Klopp N, Komulainen P, Kumari M, Langenberg C, Lehtimäki T, Lin SY, Lindström J, Loos RJF, Mach F, McArdle WL, Meisinger C, Mitchell BD, Müller G, Nagaraja R, Narisu N, Nieminen TVM, Nsubuga RN, Olafsson I, Ong KK, Palotie A, Papamarkou T, Pomilla C, Pouta A, Rader DJ, Reilly MP, Ridker PM, Rivadeneira F, Rudan I, Ruokonen A, Samani N, Scharnagl H, Seeley J, Silander K, Stančáková A, Stirrups K, Swift AJ, Tiret L, Uitterlinden AG, van Pelt LJ, Vedantam S, Wainwright N, Wijmenga C, Wild SH, Willemsen G, Wilsgaard T, Wilson JF, Young EH, Zhao JH, Adair LS, Arveiler D, Assimes TL, Bandinelli S, Bennett F, Bochud M, Boehm BO, Boomsma DI, Borecki IB, Bornstein SR, Bovet P, Burnier M, Campbell H, Chakravarti A, Chambers JC, Chen YDI, Collins FS, Cooper RS, Danesh J, Dedoussis G, de Faire U, Feranil AB, Ferrières J, Ferrucci L, Freimer NB, Gieger C, Groop LC, Gudnason V, Gyllensten U, Hamsten A, Harris TB, Hingorani A, Hirschhorn JN, Hofman A, Hovingh GK, Hsiung CA, Humphries SE, Hunt SC, Hveem K, Iribarren C, Järvelin MR, Jula A, Kähönen M, Kaprio J, Kesäniemi A, Kivimaki M, Kooner JS, Koudstaal PJ, Krauss RM, Kuh D, Kuusisto J, Kyvik KO, Laakso M, Lakka TA, Lind L, Lindgren CM, Martin NG, März W, McCarthy MI, McKenzie CA, Meneton P, Metspalu A, Moilanen L, Morris AD, Munroe PB, Njølstad I, Pedersen NL, Power C, Pramstaller PP, Price JF, Psaty BM, Quertermous T, Rauramaa R, Saleheen D, Salomaa V, Sanghera DK, Saramies J, Schwarz PEH, Sheu WHH, Shuldiner AR, Siegbahn A, Spector TD, Stefansson K, Strachan DP, Tayo BO, Tremoli E, Tuomilehto J, Uusitupa M, van Duijn CM, Vollenweider P, Wallentin L, Wareham NJ, Whitfield JB, Wolffenbuttel BHR, Altshuler D, Ordovas JM, Boerwinkle E, Palmer CNA, Thorsteinsdottir U, Chasman DI, Rotter JI, Franks PW, Ripatti S, Cupples LA, Sandhu MS, Rich SS, Boehnke M, Deloukas P, Mohlke KL, Ingelsson E, Abecasis GR, Daly MJ, Neale BM, Kathiresan S (2013). Common variants associated with plasma triglycerides and risk for coronary artery disease. Nat Genet.

[20] Goedeke L, Rotllan N, Canfrán-Duque A, Aranda JF, Ramírez CM, Araldi E, Lin CS, Anderson NN, Wagschal A, de Cabo R, Horton JD, Lasunción MA, Näär AM, Suárez Y, Fernández-Hernando C (2015). Identification of miR-148a as a novel regulator of cholesterol metabolism. Nat Med.

[21] Su B, Fu Y, Liu Y, Wu H, Ma P, Zeng W (2018). Potential application of MicroRNA profiling to the diagnosis and prognosis of HIV-1 infection. Front Microbiol.

[22] Balasubramaniam M, Pandhare J, Dash C (2018). Are microRNAs important players in HIV-1 infection? An Update. Viruses.

[23] da Cunha J, Maselli LMF, Stern ACB, Spada C, Bydlowski SP (2015). Impact of antiretroviral therapy on lipid metabolism of human immunodeficiency virus-infected patients: old and new drugs. World J Virol.

[24] El-Sadr WM, Mullin CM, Carr A, Gibert C, Rappoport C, Visnegarwala F (2005). Effects of HIV disease on lipid, glucose and insulin levels: results from a large antiretroviral-naive cohort. HIV Med.

[25] Seedat YK (1999). Hypertension in black south Africans. J Hum Hypertens.

[26] Fourie CMT, Van Rooyen JM, Kruger A, Schutte AE (2010). Lipid abnormalities in a never-treated HIV-1 subtype C-infected African population. Lipids..

[27] Nishijima T, Shimbo T, Komatsu H, Hamada Y, Gatanaga H, Kikuchi Y, Oka S (2014). Cumulative exposure to ritonavir-boosted atazanavir is associated with cholelithiasis in patients with HIV-1 infection. J Antimicrob Chemother.

[28] Lin K-Y, Liao S-H, Liu W-C, Cheng A, Lin S-W, Chang S-Y (2015). Cholelithiasis and Nephrolithiasis in HIV-Positive Patients in the Era of Combination Antiretroviral Therapy. De Socio GV, editor. PLoS One.

[29] Verdon R, Daudon M, Albessard F, Brefort JL, Bazin C (2002). Indinavir-induced cholelithiasis in a patient infected with human immunodeficiency virus. Clin Infect Dis.

[30] Lake JE, Currier JS (2013). Metabolic disease in HIV infection. Lancet Infect Dis.

[31] Livak KJ, Schmittgen TD (2001). Analysis of relative gene expression data using real-time quantitative PCR and the 2-ΔΔCT method. Methods..

[32] Kern F (1994). Effects of dietary cholesterol on cholesterol and bile acid homeostasis in patients with cholesterol gallstones. J Clin Invest.

[33] Atamanalp SS, Keles MS, Atamanalp RS, Acemoglu H, Laloglu E (2013). The effects of serum cholesterol, LDL, and HDL levels on gallstone cholesterol concentration. Pak J Med Sci.

[34] Lee DWT, Gilmore CJ, Bonorris G, Cohen H, Marks JW, Cho-Sue M, Meiselman MS, Schoenfield LJ (1985). Effect of dietary cholesterol on biliary lipids in patients with gallstones and normal subjects. Am J Clin Nutr.

[35] Assmann G, Gotto Jr AM. HDL cholesterol and protective factors in atherosclerosis. Circulation. 2004; 109(23_suppl_1):3–8. 10.1161/01.CIR.0000131512.50667.46 15198960

[36] Juvonen T, Savolainen MJ, Kairaluoma MI, Lajunen LH, Humphries SE, Kesäniemi YA (1995). Polymorphisms at the apoB, apoA-I, and cholesteryl ester transfer protein gene loci in patients with gallbladder disease. J Lipid Res.

[37] Hayes KC, Livingston A, Trautwein EA (1992). Dietary impact on biliary lipids and gallstones. Annu Rev Nutr.

[38] Anastos K, Lu D, Shi Q, Tien PC, Kaplan RC, Hessol NA, Cole S, Vigen C, Cohen M, Young M, Justman J (2007). Association of serum lipid levels with HIV serostatus, specific antiretroviral agents, and treatment regimens. JAIDS J Acquir Immune Defic Syndr.

[39] Riddler SA, Smit E, Cole SR, Li R, Chmiel JS, Dobs A, Palella F, Visscher B, Evans R, Kingsley LA (2003). Impact of HIV infection and HAART on serum lipids in men. Jama..

[40] Yokoyama C, Wang X, Briggs MR, Admon A, Wu J, Hua X, Goldstein JL, Brown MS (1993). SREBP-1, a basic-helix-loop-helix-leucine zipper protein that controls transcription of the low density lipoprotein receptor gene. Cell..

[41] Brown MS, Goldstein JL (1997). The SREBP pathway: regulation of cholesterol metabolism by proteolysis of a membrane-bound transcription factor. Cell..

[42] Taylor HE, Linde ME, Khatua AK, Popik W, Hildreth JEK (2011). Sterol regulatory element-binding protein 2 couples HIV-1 transcription to cholesterol homeostasis and T cell activation. J Virol.

[43] Petit JM, Duong M, Duvillard L, Florentin E, Portier H, Lizard G, Brun JM, Gambert P, Verges B (2002). LDL-receptors expression in HIV-infected patients: relations to antiretroviral therapy, hormonal status, and presence of lipodystrophy. Eur J Clin Investig.

[44] Vaisman BL, Lambert G, Amar M, Joyce C, Ito T, Shamburek RD, Cain WJ, Fruchart-Najib J, Neufeld ED, Remaley AT, Brewer HB, Santamarina-Fojo S (2001). ABCA1 overexpression leads to hyperalphalipoproteinemia and increased biliary cholesterol excretion in transgenic mice. J Clin Invest.

[45] Lee J, Tauscher A, Seo DW, Oram JF, Kuver R (2003). Cultured gallbladder epithelial cells synthesize apolipoproteins AI and E. Am J Physiol Liver Physiol.

[46] Vickers KC, Shoucri BM, Levin MG, Wu H, Pearson DS, Osei-Hwedieh D, Collins FS, Remaley AT, Sethupathy P (2013). MicroRNA-27b is a regulatory hub in lipid metabolism and is altered in dyslipidemia. Hepatology..

[47] Bayly GR. Lipids and disorders of lipoprotein metabolism. In: Clinical Biochemistry: Metabolic and Clinical Aspects. Elsevier. 2014:702–36.

[48] Sopeyin A, Zhou L, Li M, Barakat L, Paintsil E (2019). Dysregulation of sterol regulatory element-binding protein 2 gene in HIV treatment-experienced individuals. PLoS One.

[49] Li M, Sopeyin A, Paintsil E (2018). Combination of tenofovir and emtricitabine with efavirenz does not moderate inhibitory effect of efavirenz on mitochondrial function and cholesterol biosynthesis in human T lymphoblastoid cell line. bioRxiv.

[50] Pokora Rodak A, Krzowska-Firych J, Tomasiewicz K. Concentration of LDLR, degree of hepatic fibrosis and hepatic steatosis in patients with chronic hepatitis B infection treated with tenofovir disoproxil fumarate. Ann Agric Environ Med. 2020. 10.26444/aaem/122627. 10.26444/aaem/122627 34558270

